# Case Report: Diagnostic difficulties, treatment, and association of the MEFV E148Q variant in a patient with PASH syndrome

**DOI:** 10.3389/fmed.2025.1557540

**Published:** 2025-08-08

**Authors:** Qianfeng Qin, Cunwei Cao, Jiarong Liang, Bingkun Li, Jiaguang Su

**Affiliations:** ^1^Department of Dermatology and Venereology, The First Affiliated Hospital of Guangxi Medical University, Nanning, Guangxi, China; ^2^Fangchenggang Wanqing Institute of Mycosis Prevention and Control, Fangchenggang, Guangxi, China; ^3^Guangxi Key Laboratory of Mycosis Prevention and Treatment, Nanning, Guangxi, China

**Keywords:** PASH syndrome, pyoderma gangrenosum, acne, suppurative hidradenitis, *MEFV* variant, E148Q, adalimumab

## Abstract

**Background:**

PASH syndrome is a rare autoinflammatory disorder characterized primarily by pyoderma gangrenosum, acne, and suppurative hidradenitis. It is frequently misdiagnosed or underdiagnosed due to its diverse clinical manifestations. PASH syndrome is considered a polygenic autoinflammatory disease associated with multiple gene variants, among which *MEFV* variants may play a significant role. This case report describes a PASH syndrome patient with the *MEFV* variant (NM_000243.3.442G > C; p.Glu148Gln). The E148Q variant appears with high frequency as a homozygote in the gnomAD database, and its pathogenicity remains controversial.

**Case presentation:**

This case report describes a Chinese male patient with PASH syndrome who initially presented with severe acne and suppurative hidradenitis that were unresponsive to conventional therapy, subsequently developing pyoderma gangrenosum, leading to the diagnosis of PASH syndrome. Whole-exome sequencing performed during the diagnostic workup revealed the *MEFV* gene variant (p.E148Q). Treatment with adalimumab ultimately achieved favorable therapeutic outcomes.

**Conclusion:**

This case report represents the first documentation of a PASH syndrome patient carrying the homozygous E148Q variant. Adalimumab demonstrated good therapeutic efficacy; however, disease recurrence occurred due to poor medication adherence, highlighting the importance of long-term management. The pathogenicity of E148Q alone is relatively weak and likely depends on polygenic accumulation or epigenetic dysregulation. It may contribute to disease pathogenesis through a “variant load” mechanism in conjunction with ethnic differences and other genetic or environmental factors.

## 1 Introduction

PASH syndrome (pyoderma gangrenosum, acne, and suppurative hidradenitis) is a rare and complex autoinflammatory disease encompassing three main clinical manifestations: pyoderma gangrenosum (PG), acne, and hidradenitis suppurativa (HS). Since Markus Braun-Falco first proposed PASH syndrome in 2011, its diverse clinical presentations have often been overlooked by clinicians, leading to misdiagnosis or delayed diagnosis (1, 2).

Currently, PASH syndrome is considered a polygenic autoinflammatory disease. Multiple gene variants have been associated with this condition, including those in *NCSTN*, *PSENEN*, *NOD2*, *PSTPIP1*, *NLRC4*, *OTULIN*, *GJB2* and *MEFV* (p.I591T) (3, 4). A study investigating genetic variants associated with hidradenitis suppurativa (HS) identified two predominant variants significantly correlated with HS: rs10512572 located on chromosome 17 near the *SOX9* gene, and rs17090189 located on chromosome 13 near the *KLF5* gene (5), which may be associated with PASH syndrome. Among these, *MEFV* gene variants are closely associated with familial Mediterranean fever (FMF), which is aa classic monogenic autoinflammatory disease (6). Studies have demonstrated elevated *MEFV* variant rates in patients with complex HS (including PASH syndrome), potentially playing an important role in autoinflammatory pathogenesis (7). The *MEFV* (p.E148Q) variant identified in this case has been previously reported in patients with PG and familial Mediterranean fever (FMF), but its role in PASH syndrome remains unclear (8, 9). In previous reports, the genetic status of E148Q includes heterozygous, compound heterozygous, and homozygous states. Additionally, this variant appears with high frequency in homozygous form in the gnomAD database. Its pathogenicity remains controversial: traditionally, scholars have considered E148Q a variant of uncertain significance (10); however, other studies have published different perspectives, with one study showing no statistical differences in symptoms among heterozygous, compound heterozygous, and homozygous patients, concluding that E148Q is a disease-causing variant (9).

This study reports a challenging case of PASH syndrome in which whole exome sequencing detected the *MEFV* gene variant (p.E148Q). This represents the first reported case of this variant identified in a patient with PASH syndrome.

## 2 Case presentation

**Figure 1:**
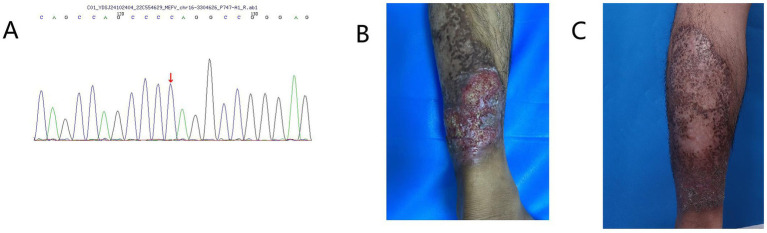
**(A)** Whole-exome sequencing identified a NM_000243.3.442G > C (p.E148Q) variant of the *MEFV* gene in the patient. **(B)** Before adalimumab treatment, the patient had a non-healing ulcerative lesion on the left lower limb. **(C)** After treatment with adalimumab, the patient’s skin lesions on the left lower limb healed.

**Figure 2:**
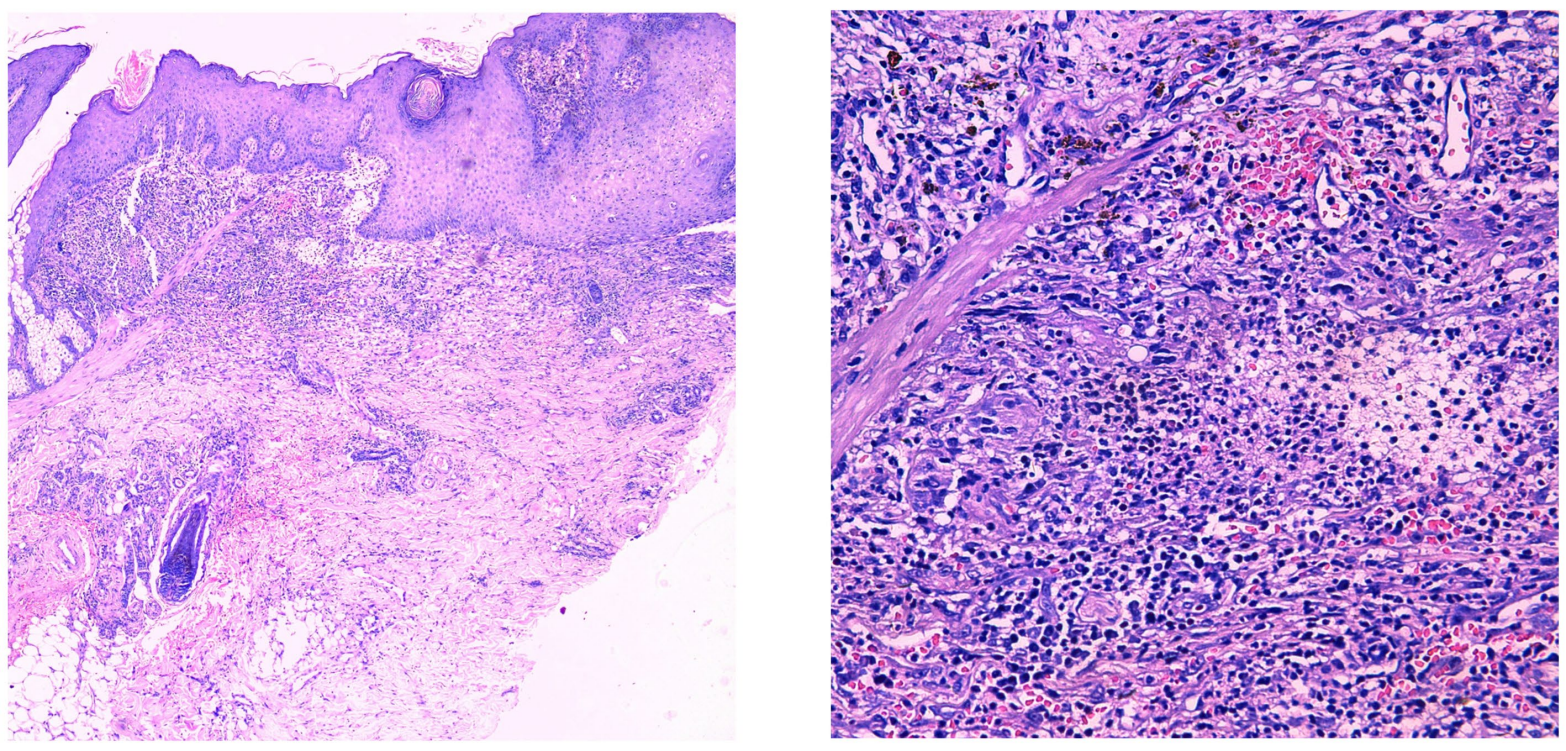
Pseudoepitheliomatous hyperplasia of the epidermis with extensive lymphocytic and neutrophilic infiltration in the superficial to mid-dermis.

The patient is a 25-year-old Asian male residing in southern China, working as a truck driver, with a 6-year smoking history and no family history of pyoderma gangrenosum (PG), severe acne, hidradenitis suppurativa (HS), or familial Mediterranean fever (FMF). In July 2021, the patient presented to our hospital with erythema, papules, and nodules on the head and face accompanied by pruritus, which gradually spread to the trunk, extremities, and axillae, without fever, abdominal pain, chest pain, or joint pain. The presentation met diagnostic criteria for severe acne and hidradenitis suppurativa. Despite standard treatment, therapeutic response was suboptimal, causing significant psychological distress for the patient. In November 2022, the patient developed extensive erythema and nodules on the left lower extremity, which gradually progressed to ulcerative lesions (Figure 1). Histopathological examination confirmed the diagnosis of pyoderma gangrenosum (Figure 2).

Initial treatment with prednisolone acetate resulted in partial ulcer control but incomplete healing. Self-discontinuation of medication led to re-expansion of lesions. Adalimumab therapy initiated in August 2023 resulted in significant improvement of lesions with marked ulcer reduction; however, due to irregular follow-up, disease recurrence occurred after medication discontinuation. The recurrent exacerbations caused the patient extreme physical and psychological distress. Based on medical history and clinical presentation, a final diagnosis of PASH syndrome was established. To exclude other genetic disorders and elucidate the genetic basis of PASH syndrome, we performed whole-exome sequencing and analysis on the patient at no charge. The homozygous *MEFV* variant (E148Q) was identified (Figure 1), while the aforementioned variants rs10512572 and rs17090189, as well as genetic variants in *NCSTN*, *PSENEN*, *NOD2*, *PSTPIP1*, *NLRC4*, *OTULIN*, *GJB2*, and *MEFV* (p.I591T), were not detected. Unfortunately, due to family members being occupied with work, no familial segregation study was conducted. During treatment, recurrent lesions and treatment resistance presented significant clinical management challenges. Disease control was ultimately achieved through a combination regimen of adalimumab with antibiotics and corticosteroids. The patient expressed satisfaction with the current treatment outcomes.

## 3 Discussion and conclusion

Current literature indicates that PASH syndrome exhibits highly heterogeneous clinical manifestations, with no unified diagnostic and therapeutic guidelines currently available (4). This patient’s therapeutic response to adalimumab aligns with previous reports, suggesting potential efficacy of this medication for PASH syndrome (11). However, poor medication adherence and irregular follow-up led to multiple disease recurrences, highlighting the importance of long-term medication management and ensuring good patient compliance (12).

A recent study found that in FMF patients, the presence of multiple variants may be associated with higher occurrence rates of moderate disease severity (13). Another study found that isolated *MEFV* variants result in limited IL-1β secretion increase, requiring cooperation with other variants to reach the pathogenic threshold for FMF (14). Both studies support the “variant burden” hypothesis. Therefore, E148Q may act in conjunction with multiple other moderate/high-frequency variants (polygenic risk) rather than as a single potent pathogenic variant. Additionally, there may be many incorrectly diagnosed patients, which together with variant burden contributes to the high frequency phenomenon of 5,896 homozygotes for the *MEFV* gene c.442G > C (p.Glu148Gln) variant in the gnomAD database.

Recent scholars have discovered that among FMF patients, whether p.E148Q/p.E148Q or p.E148Q/p.E148Q + V726A, Druze individuals comprise a higher proportion compared to other ethnic groups, suggesting a connection between E148Q variant pathogenicity and ethnicity (15). In this PASH syndrome patient, although whole exome sequencing only detected homozygous *MEFV* (p.E148Q) variant, considering the clinical complexity and the relatively weak pathogenicity of this *MEFV* variant, there may be undiscovered synergistic variants (such as non-coding variants, epigenetic regulatory abnormalities, or polygenic cumulative effects). Certain exons of some genes (such as *NCSTN*, *PSENEN*) may not have been effectively captured due to high GC content or complex structures. Furthermore, smoking may exacerbate the pro-inflammatory effects of E148Q through epigenetic silencing of anti-inflammatory genes (such as IL-10) (16). These factors may explain why the patient ultimately presented with PASH rather than typical FMF. Whether E148Q is the primary driving factor or merely serves as a modifier of inflammatory response requires more comprehensive genomic analysis and functional experiments to elucidate the complete mechanism.

This study has several limitations. First, as a single-case analysis without familial segregation studies, establishing definitive genotype–phenotype associations remains challenging. Second, whole-exome sequencing has inherent limitations, being unable to detect potentially pathogenic variants in non-coding regulatory regions and intronic sequences. Additionally, although we identified the *MEFV* E148Q variant, and previous studies suggest that *MEFV* variants may cause neutrophil dysfunction (6) (providing important insights into the pathogenesis of PASH syndrome), our study lacks functional validation to determine whether this variant contributes to disease development through effects on neutrophil function. Future studies should include larger cohorts and multicenter collaborations to address these limitations, while conducting more comprehensive experimental research to clarify the pathogenic mechanisms of the E148Q variant in PASH syndrome.

## Data Availability

The whole-exome sequencing data generated in this study have been deposited in the NCBI Sequence Read Archive (SRA) under BioProject accession number PRJNA1297302.
